# Impact of opportunistic screening on squamous cell and adenocarcinoma of the cervix in Germany: A population-based case-control study

**DOI:** 10.1371/journal.pone.0253801

**Published:** 2021-07-14

**Authors:** Luana F. Tanaka, Dirk Schriefer, Kathrin Radde, Gunther Schauberger, Stefanie J. Klug

**Affiliations:** 1 Chair of Epidemiology, Department of Sport and Health Sciences, Technical University of Munich, Munich, Germany; 2 Center of Clinical Neuroscience, University Clinic Carl Gustav Carus Dresden, Dresden, Germany; University of Zurich, SWITZERLAND

## Abstract

**Background:**

We investigated the uptake of opportunistic cervical cancer screening (CCS) and other risk factors and their association with cervical cancer in Germany in a case-control study.

**Methods and findings:**

We recruited incident cases of cervical cancer (ICD-10 C53) diagnosed between 2012 and 2016 and matched with three population-based controls, based on age and region of residence. Cases and controls reported their CCS participation during the past ten years (frequent: every three years; no or infrequent: less than every three years) and other relevant variables. We fitted conditional logistic regression models, reporting odds ratios (OR) and 95% confidence intervals (95% CI). We report overall and stratified analyses by histologic group (squamous cell–SCC, and adenocarcinoma–AC), T category (T1 and T2+), and age (<50 and ≥50 years). We analysed 217 cases and 652 matched controls. 53.0% of cases and 85.7% of controls attended CCS frequently. In the overall adjusted model, no or infrequent participation in CCS (OR 5.63; 95% CI 3.51 to 9.04), having had more than one sexual partner (OR 2.86; 95%CI 1.50 to 5.45) and obesity (OR 1.69; 95% CI 1.01 to 2.83) were associated with cervical cancer. Twelve years of schooling (OR 0.37; 95% CI 0.23 to 0.60) and a net monthly income of €3000 or more (OR 0.50; 95% CI 0.30 to 0.82) were protective factors. In the stratified analyses, no or infrequent participation was associated with T1 (OR 4.37; 95% CI 2.48 to 7.71), T2+ (OR 10.67; 95% CI 3.83 to 29.74), SCC (OR 6.88; 95% CI 4.08 to 11.59) and AC (OR 3.95; 95% CI 1.47 to 10.63).

**Conclusion:**

Although women who frequently attended CCS were less likely to develop cervical cancer, especially larger tumours, the high proportion of cases who had been frequently screened prior to diagnosis underscores the need to investigate the quality of cytology and treatment of precancerous lesions in Germany.

## Introduction

Although the incidence of cervical cancer in Germany has declined for decades, it still remains slightly higher in comparison to other countries in Western Europe (8.8 per 100 000 women, European standard). In 2020, 4666 new cases and 2075 deaths from cervical cancer occurred, representing age-standardised incidence and mortality rates of 9.6 and 4.2 per 100 000 women, respectively [1].

Infection with high-risk human papillomavirus (HPV) is a necessary but not a sufficient cause for the development of cervical cancer. Other factors, including the number of sexual partners, immune status, smoking, use of oral contraceptives, other sexually transmitted infections, and parity, may contribute to cervical carcinogenesis [2]. Additionally, infrequent attendance in cervical cancer screening (CCS) has been described as an important risk factor, as CCS can prevent invasive cancer by detecting precancerous lesions [3]. A meta-analysis of studies conducted in several high-income countries showed that 53.8% of women diagnosed with cervical cancer had inadequate screening histories regarding the recommended screening intervals [4].

From 1971 to 2020, all women in Germany from the age of 20 were entitled to an opportunistic annual cytological screening examination free of costs. Conventional cytology was predominantly used, but only in 2005, German authorities recommended the use of speculum and brush to collect cervical smears [5]. The estimated triennial participation rate ranges from 75% to 85% [6–8]. Data on the association between CCS participation and cervical cancer in Germany are limited to case series and reports but indicate that participation in CCS has been low among women who developed this malignancy [9]. Therefore, the present study aimed to investigate the impact of opportunistic CCS on cervical cancer, adjusting for known and potential risk factors including socio-demographic characteristics, smoking, oral contraceptive (OC) use, parity, body mass index (BMI), and physical activity.

## Materials and methods

The present analyses are based on data from the multicentre TeQaZ study (original title in German "Fall-Kontroll-Studie zur Häufigkeit der **Te**ilnahme an der Krebsvorsorge und zur **Q**u**a**lität der **Z**ytologie"), a population-based case-control study (S1 Fig). Incident cases of cervical cancer (ICD-10 C53), diagnosed between 2012 and 2016 in the German states of Saxony, Rhineland-Palatinate, and the neighbouring regions of Baden-Württemberg, Hesse, North Rhine-Westphalia, Saarland, Brandenburg, Saxony-Anhalt, and Thuringia were recruited via hospital departments of gynaecology. Before the recruitment phase, the collaborating hospitals were informed about the study and its requirements. Study information and documentation materials were provided.

Women newly diagnosed with cervical cancer were informed about the TeQaZ study by the oncologist while in the hospital. Those who agreed to participate were given information material, including a cover letter, a study brochure, and an informed consent form. The signed consent form, the completed documentation, and the histological confirmation of the C53 diagnosis were sent to the study centre by the hospitals.

Cases were matched with three population-based controls, recruited via population registries, based on age (+/-2 years of birth date) and region of residence, which provided the name, date of birth, and the address of the potential controls to the study centre. Potential controls were sent a cover letter inviting them to join the study and, additionally, a study brochure and an informed consent form. They had to return the signed informed consent and provide their telephone number for a telephone pre-screen and interview in case of eligibility. The study received ethical approval from all states where it was conducted.

### Inclusion and exclusion criteria

All women diagnosed with invasive cervical cancer (ICD-10 C53) in the study regions and reported to the study centre during the recruitment phase (October 2012 to June 2016) were considered eligible for inclusion as cases. Controls were excluded if they had been previously diagnosed with invasive cervical cancer or had undergone a hysterectomy, while those with carcinoma *in situ* (ICD-10 D06) were eligible for inclusion. The eligibility of potential controls was assessed during a pre-screen interview.

### Data collection

The cases and controls were contacted at home by trained interviewers to complete a computer-assisted telephone interview (Voxco Montreal, Quebec, Canada) using a structured questionnaire. During the interview (median duration 18.0 minutes, mean duration 19.5 minutes), the women were asked in detail about their participation in CCS, year by year, in the past ten years, as well as their lifetime attendance. Socio-demographics and other variables were also collected, including education, income, whether they were living with a partner, parity, OC use, number of sexual partners, history of herpes, chlamydia, and condyloma, smoking, BMI (calculated from self-reported weight in kilograms and height in meters), physical activity (any bodily movement, including daily activities such as walking to work or gardening), sport (any planned, structured, and repetitive activity to improve or maintain physical fitness), and fruit and vegetable intake. Interviewers were blinded to the case/control status of the women.

Tumour information, including histology, cell grading, and staging, were collected from the hospital records of the cases. Tumours were classified by histology as squamous cell carcinoma (SCC), adenocarcinoma/adenosquamous carcinoma (AC), or other.

### Non-participation in the TeQaZ study

Eligible women who did not want to participate in the study were asked to answer a short questionnaire containing socio-demographic information anonymously.

### Statistical analyses

Participation in CCS was classified as frequent if women had been to CCS at least every three years within the past ten years, including at least once in the three years preceding diagnosis/study inclusion, or no/infrequent, in case attendance was less regular. We excluded one case and two controls from our analyses who reported zero sexual partner.

We conducted conditional logistic regression analyses to assess the association between CCS participation and cervical cancer. The overall multivariable model was adjusted for all variables and age. We conducted additional analyses for tumour size as measured by T (T1 and T2+) for histological group (SCC and AC) and for women aged 50 and older. In addition, we conducted sensitivity analysis by excluding women younger than 30 years of age.

The results were reported as odds ratios (OR) and corresponding 95% confidence intervals (95% CI), both for controls as reference as well as cases as reference (presented in the supporting information file only) to allow for comparison with previous studies. Multiple imputation was employed for missing data in the univariable and multivariable analyses, and results were pooled based on Rubin’s rule. Analyses were performed in R with anonymised data using the packages *survival* for conditional logistic regression and *mice* for multiple imputation, respectively.

## Results

### Recruitment of gynaecological units

In total, 153 gynaecological units in the study region and the neighbouring areas were contacted. Of these, 38 (24.8%) were not eligible as they did not treat invasive cervical cancer, 31 (20.3%) did not want to participate and 2 (1.3%) did not respond. 82 of 115 eligible hospitals (71.3%) participated, 35 (42.7%) in the state of Saxony, 23 (28.1%) in Rhineland-Palatinate, and 24 (29.2%) in the neighbouring German states of North Rhine-Westfalia (n = 8), Baden-Württemberg (n = 5), Hesse (n = 3), Saarland (n = 1) (all four states bordering Rhineland-Palatinate); and Thuringia (n = 5), Saxony-Anhalt (n = 1) and Brandenburg (n = 1) (all three states bordering Saxony).

### Socio-demographic characteristics

**Fig 1:**
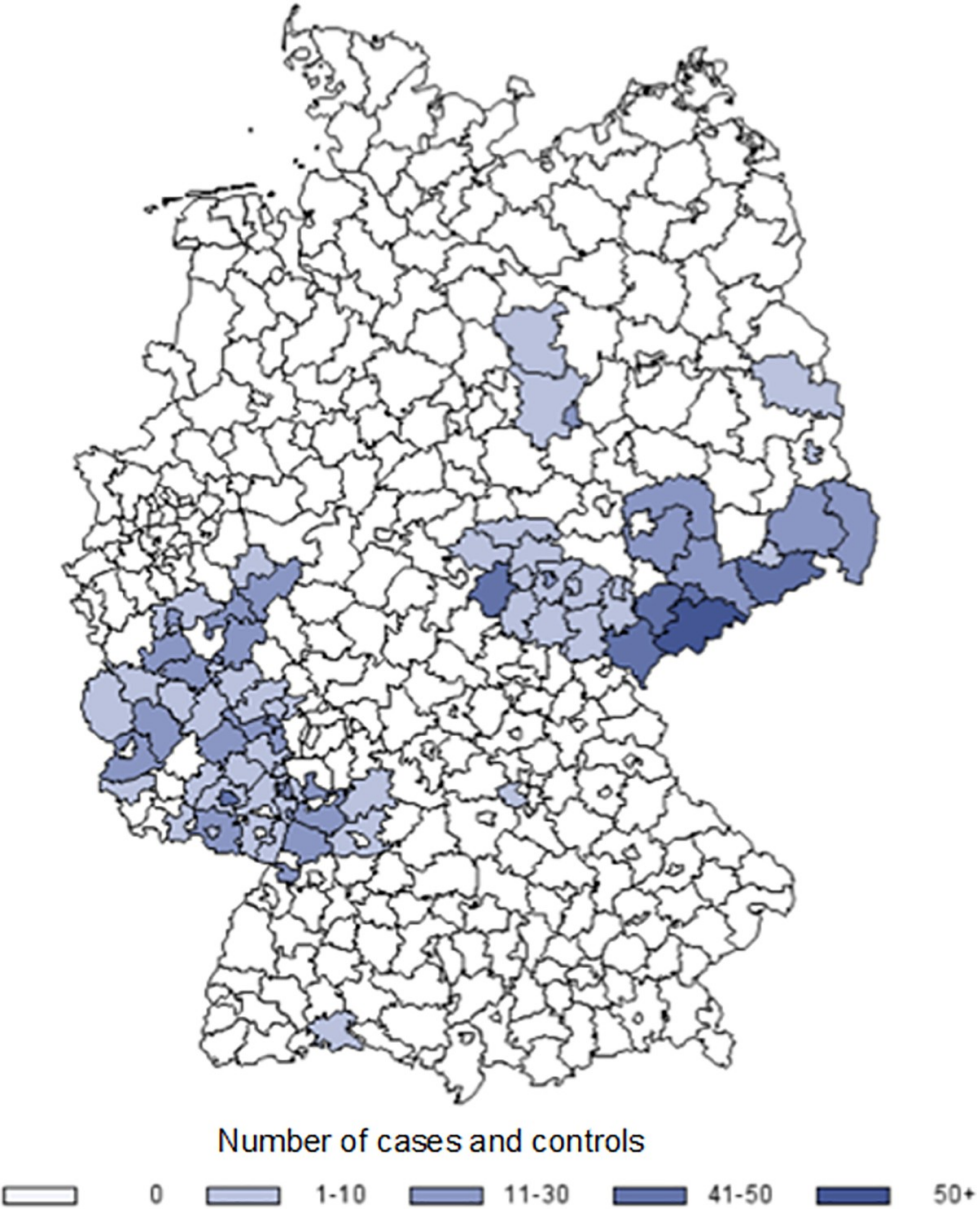
Distribution of cases and controls by region.

A total of 244 cases and 1161 controls were interviewed. Of these, 217 cases and 652 matched controls were included in the analysis (S2 Fig). Among the 869 participants, 32.9% were residents of Saxony, 40.7% from Rhineland Palatinate, and 26.4% from the neighbouring federal states (Fig 1). About 60% of women were aged 30 to 49 years (Table 1). Due to successful matching, the age and regional distribution among cases and controls did not differ.

**Table 1:** Socio-demographic characteristics and risk factors of the study population (217 cases and 652 controls).

Variable	Cases		Controls	
	n	%	n	%
Age (years)*				
20–29	6	2.8	15	2.3
30–39	56	25.8	161	24.7
40–49	79	36.4	236	36.2
50–59	38	17.5	125	19.2
60–69	32	14.7	92	14.1
70–79	6	2.8	23	3.5
Place of residence*				
Saxony	71	32.7	215	33.0
Rhineland-Palatinate	88	40.6	266	40.8
Neighbouring Federal States	58	26.7	171	26.2
**Nationality**				
German	209	96.3	636	97.5
Other	8	3.7	16	2.5
**Education (years)**				
≤ 9 years	40	18.4	50	7.7
10 years	118	54.4	308	47.2
≥ 12 years	53	24.4	294	45.1
Missing	6	2.8	0	0.0
**Net monthly household income (€)**				
< 3000	143	65.9	282	43.3
≥ 3000	46	21.1	288	44.2
Missing	28	12.9	82	12.6
**Currently living with a partner**				
Yes	170	78.3	570	87.4
No	46	21.2	82	12.6
Missing	1	0.5	0	0.0
**Parity**				
0	44	20.3	113	17.3
1	62	28.6	175	26.8
2–3	93	42.9	342	52.5
4 or more	18	8.3	22	3.4
**Ever use of oral contraceptives**				
Yes	179	82.5	592	90.8
No	32	14.7	60	9.2
Missing	6	2.8	0	0.0
**Number of sexual partners**				
1	18	8.3	134	20.6
2–3	168	77.4	441	67.6
≥ 4	26	12.0	67	10.3
Missing	5	2.3	10	1.5
**Ever genital Herpes infection**				
Yes	8	3.7	17	2.6
No	208	95.9	631	96.8
Missing	1	0.5	4	0.6
**Ever Chlamydia infection**				
Yes	18	8.3	63	9.6
No	188	86.6	569	87.3
Missing	11	5.1	20	3.1
**Ever Condyloma infection**				
Yes	11	5.1	30	4.6
No	206	94.9	616	94.5
Missing	0	0.0	6	0.9
**Ever smoked**				
Yes	126	58.1	273	41.9
No	91	41.9	379	58.1
Missing	0	0.0	0	0.0
**Body mass index (BMI) kg/m**^**2**^				
< 18.5	8	3.7	19	2.9
18.5–24.9	104	47.9	357	54.8
25–29.9	60	27.6	196	30.1
≥ 30	45	20.7	80	12.3
**Physical activity of at least 30 minutes/day**				
Yes	206	94.5	602	92.0
No	12	5.5	51	7.8
Missing	0	0.0	1	0.2
**Sporting activity**				
Never	100	46.1	176	27.0
1–3 times/month or less	24	11.1	72	11.0
1–2 times/week	47	21.7	266	40.8
3–4 times/week	23	10.6	104	16.0
At least 5 times/week	22	10.1	34	5.2
Missing	1	0.5	0	0.0
**Number of portions of fruit and vegetables/day**				
< 3	139	64.1	371	56.9
≥ 3	76	35.0	281	43.1
Missing	2	0.9	0	0.0
**TOTAL**	217	100	652	100

*matching variables

Most study participants were German citizens. About a quarter of cases had completed 12 years of schooling and had a net monthly household income of €3000 or higher, compared to nearly half of controls. The majority of women reported currently living with a partner (78.3% of cases and 87.4% of controls), ever use of OC (82.5% of cases and 90.8% of controls), and having had more than one sexual partner (89.4% of cases and 77.9% of controls). Chlamydia was the most frequently reported sexually transmitted infection by both cases (8.3%) and controls (9.6%).

The prevalence of ever smoking in cases and controls was 58.1% and 41.9%, respectively. About a fifth of cases, as compared to 12.3% of controls were obese (BMI of 30 kg/m^2^ or higher). Most women reported engaging in some kind of physical activity at least 30 minutes a day (94.5% of cases vs 92.0% of controls).

### Pathologic characteristics of tumours (cases)

Overall, SCC accounted for 79.3% of tumours, whereas AC, including adenosquamous carcinoma, was found in 18.4% of women. Most tumours were either intermediate (43.3%) or high grade (36.4%). As for the T category, the majority of tumours (62.7%) were classified as T1, with a significantly higher proportion among those aged <50 years in comparison to older women (70.9% vs 47.4%, p<0.001) (S1 Table).

### CCS participation and its association with selected variables

In all, 43.3% of cases and 71.5% of controls had attended CCS yearly. Frequent participation (at least every three years, including at least once in the three years before diagnosis/study inclusion) was reported by 53.0% of cases and 85.7% of controls. No or infrequent attendance was reported by 47.0% and 14.3% of cases and controls, respectively (Table 2). Screening uptake among cases was similar by grading (53.3% for low/intermediate and 46.7% for high). When stratifying by T category, 36.0% and 73.3% of cases with T1 and T2+, respectively, reported infrequent or no participation (versus 14.5% and 15.7% of controls, respectively). Further stratification by the histological group revealed consistent results for SCC and AC: 71.4% and 80.0% of women with T2+ tumours had no or infrequent screening preceding their diagnosis (Table 3).

**Table 2:** Participation in cervical cancer screening during the past ten years among cases and controls (217 cases and 652 controls).

Participation in cervical cancer screening* by T category and grade**	Cases		Controls		OR (95% CI)	Adjusted OR (95% CI)***
	n	%	n	%		
**All**						
Frequent	115	53.0	559	85.7	Reference	Reference
No or infrequent	102	47.0	93	14.3	6.24 (4.18 to 9.32)	5.63 (3.51 to 9.04)
**T category**						
**T 1**						
Frequent	87	64.0	348	85.5	Reference	Reference
No or infrequent	49	36.0	59	14.5	3.74 (2.29 to 6.13)	4.37 (2.48 to 7.71)
**T 2+**						
Frequent	12	26.7	113	84.3	1	1
No or infrequent	33	73.3	21	15.7	11.93 (4.81 to 29.59)	10.67 (3.83 to 29.74)
**Grade**						
**Well and moderate**						
Frequent	56	53.3	270	85.7	Reference	Reference
No or infrequent	49	46.7	45	14.3	6.05 (3.41 to 10.74)	7.21 (3.69 to 14.11)
**Poor**						
Frequent	40	50.6	200	85.1	Reference	Reference
No or infrequent	39	49.4	35	14.9	5.94 (3.14 to 11.23)	4.64 (2.24 to 9.63)

* Frequent: at least every three years in the last ten years; infrequent: less frequently than every three years to once in the last ten years; no: no lifetime participation or no participation in the past ten years

** T category and grade apply only to cases; the proportion of controls presented corresponds to those matched to cases within these categories

*** Adjusted for education, income, number of sexual partners, body mass index and age

**Table 3:** Participation in cervical cancer screening during the past ten years among cases and controls, according to T category, squamous cell carcinoma (172 cases and 512 controls).

Participation in cervical cancer screening* by T category**	Cases		Controls		OR (95% CI)	Adjusted OR (95% CI)***
	N	%	N	%		
**All**						
Frequent	89	51.7	444	86.7	Reference	Reference
No or infrequent	83	48.3	68	13.3	7.06 (4.45 to 11.21)	6.88 (4.08 to 11.59)
**T 1**						
Frequent	67	61.5	279	85.6	Reference	Reference
No or infrequent	42	38.5	47	14.4	4.36 (2.49 to 7.62)	4.99 (2.62 to 9.51)
**T 2+**						
Frequent	10	28.6	90	86.5	Reference	Reference
No or infrequent	25	71.4	14	13.5	13.87 (4.57 to 42.06)	12.05 (3.40 to 42.71)

* Frequent: at least every three years in the last ten years; infrequent: less frequently than every three years to once in the last ten years; none: no lifetime participation or no participation in the past ten years

** T category applies only to cases; the proportion of controls presented corresponds to those matched to cases within these categories

*** Adjusted by education, income, number of sexual partners, body mass index and age

**Fig 2:**
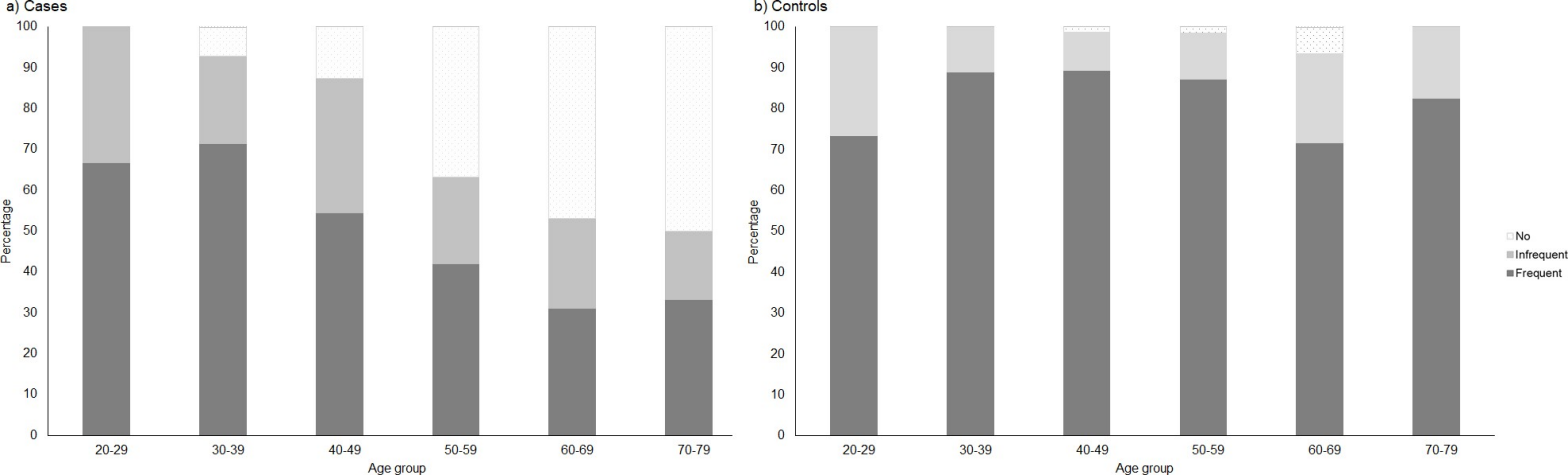
Participation in cervical cancer screening during the past ten years among cases and controls, according to age group. Frequent: at least every three years in the last ten years; infrequent: less frequently than every three years in the last ten years; no: no participation in the past ten years or no lifetime participation.

When analysing participation in CCS by age, frequent attendance was consistently observed in 70% of controls across all age groups, whereas among cases, it steadily decreased with age: from 71.0% in women aged 20 to 39 years to 31.6% among those aged 60 to 79 years (Fig 2). In this older age group, nearly half of cases (47.4%) reported no participation in CCS in the previous ten years, compared to 5.2% of controls.

Frequent CCS uptake was lower among women with nine years or less of schooling, those with a net monthly household income lower than €3000, obese women, never OC users, and those who reported engaging in sporting activity less frequently than once a week (S2 Table).

### Factors associated with cervical cancer

In the univariable analysis (Table 4), women who did not attend or infrequently attended CCS within the previous ten years were more likely to develop cervical cancer than those who participated frequently. Currently living with a partner, having at least four children, having had more than one sexual partner, ever smoking and obesity were significantly associated with cervical cancer. Having completed 12 years of schooling, a monthly net household income of €3000 or higher, ever use of OC and engaging in sporting activity at least once a week were protective.

**Table 4:** Factors associated with cervical cancer: Univariable and multivariable conditional logistic regression (217 cases and 652 controls).

Socio-demographic and risk factors	Univariable		Multivariable*	
	OR	95% CI	OR	95% CI
No or infrequent participation vs. frequent participation in cervical cancer screening [Ref.] **	6.24	4.18 to 9.32	5.63	3.51 to 9.04
School education: ≥ 12 years vs < 12 years [Ref.]	0.35	0.24 to 0.52	0.37	0.23 to 0.60
Net monthly household income: ≥ €3000 vs. < €3000 [Ref.]	0.31	0.21 to 0.46	0.50	0.30 to 0.82
Currently living with a partner: no vs yes [Ref.]	1.83	1.23 to 2.72	0.98	0.59 to 1.64
Parity: ≥ 4 children vs. < 4 children [Ref.]	2.75	1.39 to 5.40	1.86	0.72 to 4.80
Ever use of oral contraceptives: ever vs never [Ref.]	0.56	0.35 to 0.91	0.65	0.34 to 1.26
Number of sexual partners: ≥ 1 partner vs 1 partner [Ref.]	2.90	1.69 to 4.98	2.86	1.50 to 5.45
Genital herpes: ever vs never [Ref.]	1.42	0.61 to 3.32	2.40	0.82 to 7.06
Chlamydia: ever vs never [Ref.]	0.87	0.50 to 1.62	0.91	0.44 to 1.88
Condyloma: ever vs never [Ref.]	1.08	0.53 to 2.22	1.37	0.55 to 3.39
Smoking: ever vs never [Ref.]	2.01	1.45 to 2.79	1.22	0.81 to 1.83
Body Mass Index: ≥ 30 vs. < 30 kg/m^2^ [Ref.]	1.86	1.24 to 2.78	1.69	1.01 to 2.83
Physical activity: ≥ 30 minutes/day vs. < 30 minutes/day [Ref.]	1.54	0.79 to 3.00	1.71	0.55 to 1.19
Sporting activity: ≥ once a week vs < once a week [Ref.]	0.45	0.33 to 0.62	0.81	0.75 to 3.91
≥ 3 portions of fruit and vegetables a day vs < 3 portions/day [Ref.]	0.72	0.52 to 1.00	1.09	0.72 to 1.65

OR: Odds ratio, CI: Confidence interval, Ref: Reference

* Adjusted for all variables and age

** Frequent: at least every three years in the last ten years; no or infrequent: less frequently than every three years, including women who had reported no screening participation in the last ten years and women who reported no screening in their lives

Results of the multivariable analysis showed that no or infrequent CCS participation (OR 5.63; 95% CI 3.51 to 9.04), having had more than one sexual partner (OR 2.86; 95% CI 1.50 to 5.45), and obesity (OR 1.69; 95% CI 1.01 to 2.83) remained statistically significantly associated with cervical cancer while having completed 12 years of schooling (OR 0.37; 95% CI 0.23 to 0.60) and having a monthly net household income of €3000 or more (OR 0.50; 95% CI 0.30 to 0.82) were strong protective factors.

No or infrequent participation in CCS was a major risk factor in most stratified analyses. In the analyses by T category, the adjusted ORs were 4.37 (95% CI 2.48 to 7.71) and 10.67 (95% CI 3.83 to 29.74) for T1 and T2+, respectively (Table 2); for histological group, adjusted ORs were 6.88 (95% CI 4.08 to 11.59) for SCC (Table 3) and 3.95 (95% CI 1.47 to 10.63) for AC (Table 5); for women aged 50 and older the adjusted OR was 7.60 (95% CI 3.57 to 16.19) (S3 Table).

**Table 5:** Participation in cervical cancer screening during the past ten years among cases and controls, according to T category, adenocarcinoma/adenosquamous carcinoma (40 cases and 120 controls).

Participation in cervical cancer screening* by T category**	Cases		Controls		OR (95% CI)	Adjusted OR (95% CI)***
	n	%	n	%		
**All**						
Frequent	24	60.0	100	83.3	Reference	Reference
No or infrequent	16	40.0	20	16.7	3.51 (1.46 to 8.41)	3.95 (1.47 to 10.63)
**T 1**						
Frequent	19	76.0	63	84.0	Reference	Reference
No or infrequent	6	24.0	9	12.0	1.68 (0.51 to 5.54)	2.52 (0.60 to 10.54)
**T 2+**						
Frequent	2	20.0	23	76.7	Reference	****
No or infrequent	8	80.0	7	23.3	8.05 (1.21 to 53.43)	

* Frequent: at least every three years in the last ten years; infrequent: less frequently than every three years to once in the last ten years; none: no lifetime participation or no participation in the past ten years

** T category applies only to cases; the proportion of controls presented corresponds to those matched to cases within these categories

*** Adjusted by education, income, number of sexual partners, body mass index and age

**** Too few cases/controls

### CCS and cervical cancer risk

After adjusting for significant variables, frequent participation in CCS reduced the odds of cervical cancer by 82% (OR 0.18; 95% CI 0.11 to 0.28) (S4 Table). This reduction was particularly important for larger tumours with 91% (OR 0.09; 95% CI 0.03 to 0.32) and for SCC with 86% (OR 0.14; 95% CI 0.08 to 0.25; S5 and S6 Tables).

### Sensitivity analysis

The results of the sensitivity analysis excluding women younger than 30 years (S7 Table) were consistent with those which included all women.

### Non-participation in the TeQaZ study

We obtained non-participation questionnaires from 862 of 2435 women, who would have been eligible to be included in the study (35.4%; S2 Fig). When comparing schooling (32.9% with ≥12 years), participation frequency in CCS in the last ten years (84.6% with frequent participation), ever smoking (39.0%), ever use of OC (78.5%) among study participants and non-participants, distributions were relatively similar. Non-participants in our study were slightly older in comparison to participants, with almost 10% being 80 years and older.

## Discussion

TeQaZ was the first population-based case-control study investigating the uptake of opportunistic screening and its association with cervical cancer in Germany. The adjusted overall, as well as the additional analyses, consistently show that no or infrequent attendance in CCS was a major independent risk factor for cervical cancer. Obesity and having had more than one sexual partner were also associated with an increased risk for this malignancy. In contrast, having completed 12 years of schooling and a net monthly household income of 3000 € or more were found to be protective.

In the present study, 53% of cases reported frequent CCS uptake in the previous ten years, meaning that they had been screened frequently at least every three years (including once within three years prior to their diagnosis), and the remaining 47% not or infrequently been screened. When stratifying by T category, only 26.7% of cases with T2+ as compared to 64.0% with T1 tumours had been frequently screened. In Germany, a case study conducted in Mecklenburg-Western Pomerania retrospectively investigated CCS participation preceding a cervical cancer diagnosis. Fifty-eight per cent of women with cervical cancer had no Pap smear taken in the five years prior to diagnosis, whilst the remaining 42% had at least one Pap smear recorded in this period [9]. These findings were based on cytological laboratories records reported to the Quality Assurance Commission of the Medical Association in Mecklenburg-Western Pomerania. As some gynaecologists may have sent their cytological slides to laboratories in the other Federal States, the CCS participation may have been underestimated, and conclusions are limited given that there was no comparison group for these analyses.

Non-adherence to CCS intervals has been identified as the main risk factor for cervical cancer in various countries with existing organised screening programmes [10–13]. In a case-control study conducted in New Zealand, Sykes et al. (2005) found that 65% of cases with SCC and 22% of controls with cervical intraepithelial neoplasia grade three (CIN3) did not have a Pap smear in the three years preceding diagnosis [11]. An analysis of the Dutch screening programme showed that 60% of women with cervical cancer had not been screened within the five years before diagnosis [12]. In Sweden, a national audit revealed that women who had missed the two previous screening rounds had a 4.14-fold risk of cervical cancer when compared to those who had been adequately screened [13]. In the present study, women with no or infrequent CCS histories had an 8-fold risk of being diagnosed with larger tumours (T2+). Similar findings have been described for UICC stage in large audit studies in Sweden and the US [13, 14].

The protective effect of cytological screening has been extensively described in the literature [3, 15]. In a meta-analysis, Peirson et al. (2013) reported a pooled OR of 0.35 (95% CI 0.30 to 0.41) for exposure to cytological screening (versus no exposure) derived from a dozen case-control studies [3]. Landy et al. (2016), in a population-based case-control study conducted within the organised programme in the United Kingdom, described strong protection (OR 0.18; 95% CI 0.16 to 0.19) of frequent CCS attendance when compared to no or infrequent attendance among women aged 35–64 years [15], similar to the findings presented by this study.

The effectiveness of CCS programmes, however, depends largely, but not solely, on their coverage of the target population [10]. Population screening coverage might, in turn, be affected by patient’s characteristics, including age, socioeconomic status, partnership status and pre-existing health conditions [16]. In the TeQaZ study, higher schooling level and income were independently associated with reduced risk of cervical cancer. Previous studies have found similar results for cervical cancer, but similar associations have been reported for several other health conditions [17]. These associations might reflect disparities across socioeconomic status levels in access to health care, as well as behavioural aspects.

With regard to women’s reproductive histories, having had more than one sexual partner was a significant risk factor in the present study. The lifetime number of sexual partners, which leads to increased exposure to HPV infection, has been consistently linked with elevated risk for cervical cancer. In a pooled analyses of 21 epidemiological studies, women who had had more than one sexual partner had a 2-fold risk of cervical cancer when compared to women who had had a single lifetime sexual partner after adjusting for age and number of full-term pregnancies [18].

A link between OC use and increased risk for cervical cancer has been reported in several [19, 20] but not all studies [21]. Iversen et al. (2017) found a significantly elevated cervical cancer risk among current OC users and recent users, but this risk was no longer significant five years after contraception discontinuation [20]. The use of diverse approaches to classify this exposure and the substantial changes in pill formulation over the years could have contributed to these heterogeneous findings. In our analysis, ever use of OC showed a protective effect (yet not significant), likely explained by its strong association with CCS uptake, given that a medical prescription, usually supplied by a gynaecologist, is mandatory for OC provision in Germany, and gynaecologists are the main CCS providers in the country. Thus, women on OC usually receive CCS on a regular basis.

The role of sexually transmitted infections, such as *Chlamydia trachomatis* and *Herpes simplex virus* 2 in the development of cervical cancer remains unclear [22, 23]. Castellsagué et al. (2014) in a nested case-control study within the European Prospective Investigation into Cancer and Nutrition (EPIC) identified seropositivity for *Herpes simplex virus* 2 and *Chlamydia trachomatis* as possible contributing factors to cervical carcinogenesis [24]. In line with two epidemiologic studies conducted in the Nordic countries [22, 23], we did not find such associations. As infection with these sexually transmitted agents can be often asymptomatic [25], their prevalence among participants based on self-report within TeQaZ could be underestimated, attenuating true associations.

In the present study, obese women had an elevated risk of cervical cancer (although the lower boundary of the CI was 1.01, which would not represent clinical relevance) and were less likely to participate frequently in CCS than non-obese women. Hence, the association between obesity and cervical cancer could be partially attributable to lower participation in CCS, as described by Maruthur et al. (2009) [26]. Several barriers to CCS have been reported by obese patients (e.g. embarrassment of weight, concern about receiving unsolicited weight loss advice) and their healthcare providers (e.g. difficulties in examining larger patients, lack of readily available adequate resources for proper care) [27]. Moreover, as recently described by Clarke et al. (2018) in a longitudinal study, the increased risk of cervical cancer is likely a consequence of underdetection of precancerous lesions in obese women. This might be due to poor sampling (e.g. inadequately sized speculum) or impaired visualisation of the cervix during colposcopy [28]. There is scant evidence of a possible direct biological pathway linking obesity and cervical cancer [29]. However, this issue requires further assessment.

In our analyses, engaging in sporting activity at least once a week showed a protective effect in the univariable analysis but did not remain statistically significant in the fully adjusted model. To date, only a limited number of studies have examined this relationship producing inconsistent results [30].

### Strengths and limitations

The study participants were selected based on strict inclusion/exclusion criteria and successfully matched by age and study region. Furthermore, they were representative of the various German regions included in the study. Our analyses were controlled for socio-demographic and other relevant exposures, including education, reproductive history, BMI, smoking and physical activity and stratified by T category, age and tumour histology.

This study also has limitations inherent to its design. Case-control studies are prone to selection and information bias. In order to quantify such bias, the age of diagnosis of cases in the TeQaZ study was compared to the age-specific distribution of cases in the German epidemiological cancer registries. Up to the age of 50, the age distributions were very similar. However, older cases, particularly those above 70 years of age at the time of diagnosis, were underrepresented in our study. As reported by referring clinicians, older women were less likely to agree to participate in the study, partly due to difficulties associated with explaining the purpose of the study to them.

Cases were also compared with epidemiological cancer registry data for tumour size at diagnosis (T). Particularly in the Federal State of Saxony, a selection of cases with lower tumour stages at diagnosis was observed. This may have been due to referring clinicians not wanting to place the additional burden of participating in a study on women with advanced-stage cancer or that these women were physically not able to participate. Due to the voluntary nature of participation in this study, such selection bias could, however, not be avoided.

Another limitation of the present study is that analyses were based on self-reported information, and the CCS participation rates described in this study could have been somewhat overestimated. However, frequent participation in CCS reported by controls (85.7%) is similar to that described (85%) in previous studies conducted in Germany [6]. In addition, this study analysed CCS regularity in the previous ten years, which was obtained by inquiring women about their participation year by year, rather than other measures such as average participation, which would be less robust.

No information on HIV infection was collected despite its known association with cervical cancer. Nevertheless, given that the HIV prevalence among women in Germany is very low (< 0.1%) [31], it is unlikely that this information would have affected our estimates. HPV vaccination status was also not obtained, but considering that only a small fraction of the women included in this study were eligible for vaccination, the lack of this information had little impact regarding our results. This was shown in the sensitivity analysis excluding women younger than 30 years.

Due to insufficient information on N (lymph node) and M (metastases) of the TNM staging system, we were not able to conduct analyses by UICC stage; instead, our analyses were based solely on T category. Moreover, there were too few AC cases, limiting the analyses for this histologic group. As CCS seems to be more effective at detecting squamous than glandular lesions [13, 32–34], analysis with a sufficient number of cases was appropriate. However, the proportion of AC cases in our study was similar to previous reports in Germany and other countries [32, 35].

## Conclusion

Findings of the TeQaZ study show that frequent participation in CCS reduced the risk for invasive cervical cancer by 80% and by 91% for larger tumours (T2+). Thus, increasing frequent participation in CCS among under-screened women is essential to improve cervical cancer control in Germany.

As of January 2020, Germany is gradually implementing an organised CCS programme to replace its opportunistic system, informing women about CCS every five years, with the last contact at 65 years. Unfortunately, there will be no true invitational system, i.e. women will not be invited according to the specific screening intervals at every year (20–34 years) and three years (35 and older) and nor a centralised approach to monitor individual participation.

Invitations will likely increase participation [6], but this alone cannot address all barriers that prevent women from frequently attending CCS. Thus, other strategies to increase and sustain optimal participation, such as offering women HPV self-sampling or actively reminding them of CCS, possibly via modern modes of communication, must be considered.

It is striking that more than half the cases developed cervical cancer despite having been cytologically screened at least once within the three years preceding diagnosis, with 43.3% with yearly participation, indicating that abnormalities have been possibly missed multiple times or mismanaged. This underscores the necessity of investigating the quality of cytology, from adequate smear collection using correct devices to the proper classification of smears, as well as the extent to which precancerous lesions are adequately monitored and treated in Germany.

## Supporting information

S1 Fig TeQaZ study design: A population-based case-control study. (TIF)

S2 Fig Participants’ flowchart. TeQaZ study.
(TIF)

S1 Table Tumour characteristics of 217 cases by age group. (DOCX)

S2 Table Participation in cervical cancer screening during the past ten years among cases and controls by selected variables (217 cases and 652 controls). (DOCX)

S3 Table Participation in cervical cancer screening during the past ten years among cases, by age group (217 cases and 652 controls). (DOCX)

S4 Table Impact of cervical cancer screening on cervical cancer, according to tumour characteristics (217 cases and 652 controls). (DOCX)

S5 Table Impact of cervical cancer screening on cervical cancer, according to T category, squamous cell carcinoma (172 cases and 512 controls). (DOCX)

S6 Table Impact of cervical cancer screening on cervical cancer, according to T category, adenocarcinoma/adenosquamous cell carcinoma (40 cases and 120 controls). (DOCX)

S7 Table Factors associated with cervical cancer: Univariable and multivariable conditional logistic regression, excluding women younger than 30 years (211 cases and 637 controls). (DOCX)
