# Supplementary figures and images for: Impact of opportunistic screening on squamous cell and adenocarcinoma of the cervix in Germany: A population-based case-control study
Source: PLoS One. 2021 Jul 14;16(7):e0253801. doi: 10.1371/journal.pone.0253801 (PMC8279357; doi:10.1371/journal.pone.0253801)

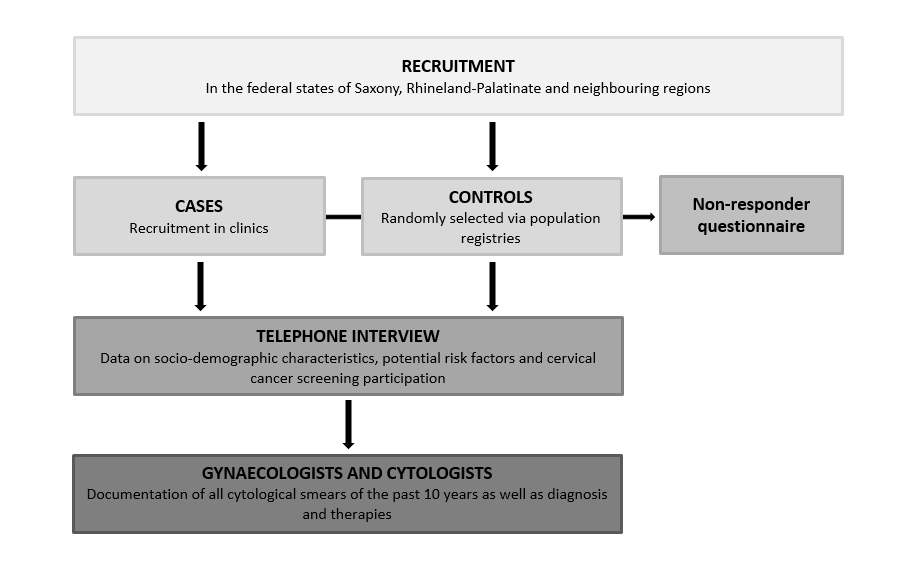

Supplement: S1 Fig — (TIF) [file pone.0253801.s001.tif]

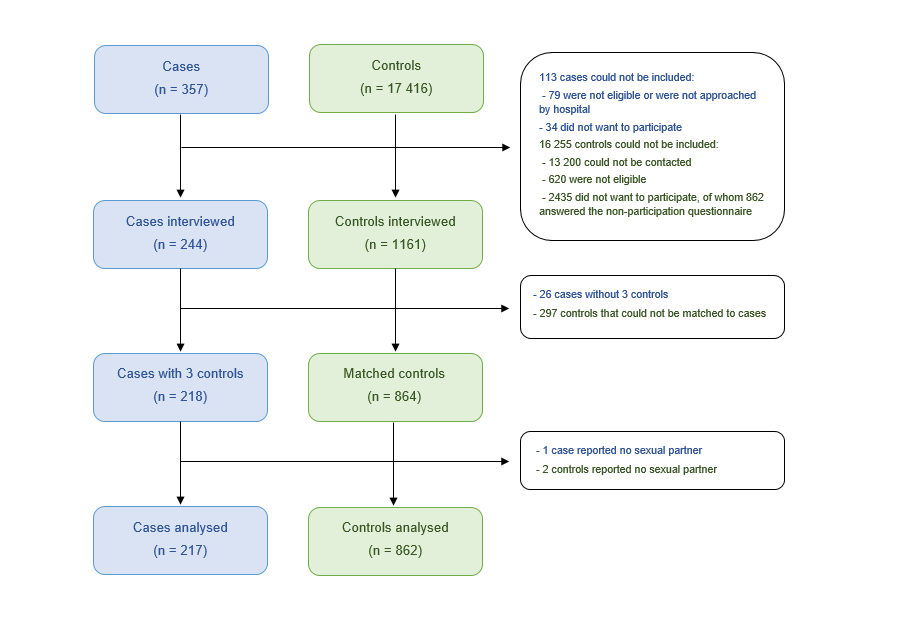

Supplement: S2 Fig — TeQaZ study. (TIF) [file pone.0253801.s002.tif]
