# Supplementary material for: Impact of opportunistic screening on squamous cell and adenocarcinoma of the cervix in Germany: A population-based case-control study
Source: PLoS One. 2021 Jul 14;16(7):e0253801. doi: 10.1371/journal.pone.0253801 (PMC8279357; doi:10.1371/journal.pone.0253801)
Supplement: S1 Table — (DOCX) [file pone.0253801.s003.docx]

**S1 Table. Tumour characteristics of 217 cases by age group**

| **Tumour characteristics** | **<50 years** | | **≥ 50 years** | | **All** | |
| --- | --- | --- | --- | --- | --- | --- |
|  | **N** | **%** | **N** | **%** | **N** | **%** |
| **Histologic group** |  |  |  |  |  |  |
| Squamous cell | 112 | 79.4 | 60 | 79.8 | 172 | 79.3 |
| Adenocarcinoma* | 25 | 17.7 | 15 | 19.7 | 40 | 18.4 |
| Missing | 4 | 2.8 | 1 | 1.3 | 5 | 2.3 |
| **T category** |  |  |  |  |  |  |
| 1 | 100 | 70.9 | 36 | 47.4 | 136 | 62.7 |
| 2 | 13 | 9.2 | 21 | 27.6 | 34 | 15.7 |
| 3+ | 6 | 4.3 | 5 | 6.6 | 11 | 5.1 |
| Missing | 22 | 15.6 | 14 | 18.4 | 36 | 16.6 |
| **Grade** |  |  |  |  |  |  |
| Low | 7 | 5.0 | 4 | 5.3 | 11 | 5.1 |
| Intermediate | 57 | 40.4 | 37 | 48.7 | 94 | 43.3 |
| High | 52 | 36.9 | 27 | 35.5 | 79 | 36.4 |
| Missing | 25 | 17.7 | 8 | 10.5 | 33 | 15.2 |

* Including adenosquamous carcinoma
