# Supplementary material for: Impact of opportunistic screening on squamous cell and adenocarcinoma of the cervix in Germany: A population-based case-control study
Source: PLoS One. 2021 Jul 14;16(7):e0253801. doi: 10.1371/journal.pone.0253801 (PMC8279357; doi:10.1371/journal.pone.0253801)
Supplement: S2 Table — (DOCX) [file pone.0253801.s004.docx]

**S2 Table. Participation in cervical cancer screening during the past ten years among cases and controls by selected variables (217 cases and 652 controls)**

|  | **Participation in cervical cancer screening*** | | | | | | | | | | | |
| --- | --- | --- | --- | --- | --- | --- | --- | --- | --- | --- | --- | --- |
|  | **Cases** | | | | | | **Controls** | | | | | |
|  | **Frequent** | | **Infrequent** | | **No** | | **Frequent** | | **Infrequent** | | **No** | |
|  | **n** | **%** | **n** | **%** | **n** | **%** | **n** | **%** | **n** | **%** | **n** | **%** |
| **Education** |  |  |  |  |  |  |  |  |  |  |  |  |
| ≤ 9 years | 17 | 42.5 | 9 | 22.5 | 14 | 35.0 | 40 | 80.0 | 10 | 20.0 | 0 | 0.0 |
| 10 years | 62 | 52.5 | 31 | 26.3 | 25 | 21.2 | 266 | 86.4 | 36 | 11.7 | 6 | 1.9 |
| ≥ 12 years | 34 | 64.2 | 12 | 22.6 | 7 | 13.2 | 253 | 86.1 | 36 | 12.2 | 5 | 1.7 |
| Missing | 2 | 33.3 | 4 | 66.7 | 0 | 0.0 | 0 | 0.0 | 0 | 0.0 | 0 | 0.0 |
| **Net monthly household income** |  |  |  |  |  |  |  |  |  |  |  |  |
| < €3 000 | 70 | 49.0 | 41 | 28.7 | 32 | 22.4 | 232 | 82.3 | 45 | 16.0 | 5 | 1.8 |
| ≥ €3 000 | 31 | 67.4 | 10 | 21.7 | 5 | 10.9 | 253 | 87.8 | 32 | 11.1 | 3 | 1.0 |
| Missing | 14 | 50.0 | 5 | 17.9 | 9 | 32.1 | 74 | 90.2 | 5 | 6.1 | 3 | 3.7 |
| **Body Mass Index (kg/m^2^)** |  |  |  |  |  |  |  |  |  |  |  |  |
| < 30 | 94 | 54.7 | 43 | 25.0 | 35 | 20.3 | 493 | 86.3 | 68 | 11.9 | 10 | 1.8 |
| ≥ 30 | 21 | 46.7 | 13 | 28.9 | 11 | 24.4 | 64 | 81.0 | 14 | 17.7 | 1 | 1.3 |
| Missing | 0 | 0.0 | 0 | 0.0 | 0 | 0.0 | 2 | 100.0 | 0 | 0.0 | 0 | 0.0 |
| **Oral contraceptive use** |  |  |  |  |  |  |  |  |  |  |  |  |
| Never | 10 | 31.3 | 7 | 21.9 | 15 | 46.9 | 51 | 85.0 | 7 | 11.7 | 2 | 3.3 |
| Ever | 104 | 58.1 | 45 | 25.1 | 30 | 16.8 | 508 | 85.8 | 75 | 12.7 | 9 | 1.5 |
| Missing | 1 | 16.7 | 4 | 66.7 | 1 | 16.7 | 0 | 0.0 | 0 | 0.0 | 0 | 0.0 |
| **Sporting activity** |  |  |  |  |  |  |  |  |  |  |  |  |
| < Once a week | 50 | 40.3 | 35 | 28.2 | 39 | 31.5 | 204 | 82.3 | 41 | 16.5 | 3 | 1.2 |
| ≥ Once a week | 65 | 70.7 | 20 | 21.7 | 7 | 7.6 | 355 | 87.9 | 41 | 10.1 | 8 | 2.0 |
| Missing | 0 | 0.0 | 1 | 100.0 | 0 | 0.0 | 0 | 0.0 | 0 | 0.0 | 0 | 0.0 |
| **TOTAL** | **115** | **53.0** | **56** | **25.8** | **46** | **21.2** | **559** | **85.7** | **82** | **12.6** | **11** | **1.7** |

* Frequent: at least every three years in the last ten years; infrequent: less frequently than every three years in the last ten years; no: no participation in the past ten years or no lifetime participation
