# Supplementary material for: Impact of opportunistic screening on squamous cell and adenocarcinoma of the cervix in Germany: A population-based case-control study
Source: PLoS One. 2021 Jul 14;16(7):e0253801. doi: 10.1371/journal.pone.0253801 (PMC8279357; doi:10.1371/journal.pone.0253801)
Supplement: S3 Table — (DOCX) [file pone.0253801.s005.docx]

**S3 Table. Participation in cervical cancer screening during the past ten years among cases, by age group (217 cases and 652 controls)**

| **Participation in cervical cancer screening* by age** | **Cases** | | **Controls** | | **OR (95% CI)** | **Adjusted OR (95% CI)**** | |
| --- | --- | --- | --- | --- | --- | --- | --- |
|  | **n** | **%** | **N** | **%** |  |  |  |
| **< 50 years** |  |  |  |  |  |  |  |
| Frequent | 87 | 61.7 | 365 | 88.6 | Reference | Reference |  |
| No or infrequent | 54 | 38.3 | 47 | 11.4 | 5.58 (3.32 to 9.40) | 5.37 (2.95 to 9.79) |  |
| **≥ 50 years** |  |  |  |  |  |  |  |
| Frequent | 28 | 36.8 | 194 | 80.8 | Reference | Reference |  |
| No or infrequent | 48 | 63.2 | 46 | 19.2 | 7.73 (3.87 to 15.43) | 7.60 (3.57 to 16.19) |  |

* Frequent: at least every three years in the last ten years; infrequent: less frequently than every three years to once in the last ten years; no: no lifetime participation or no participation in the past ten years

** Adjusted for education, income, number of sexual partners, body mass index and age
