# Supplementary material for: Impact of opportunistic screening on squamous cell and adenocarcinoma of the cervix in Germany: A population-based case-control study
Source: PLoS One. 2021 Jul 14;16(7):e0253801. doi: 10.1371/journal.pone.0253801 (PMC8279357; doi:10.1371/journal.pone.0253801)
Supplement: S4 Table — (DOCX) [file pone.0253801.s006.docx]

**S4 Table. Impact of cervical cancer screening on cervical cancer, according to tumour characteristics (217 cases and 652 controls)**

| **Participation in cervical cancer screening* by T category and grade**** | **Cases** | | **Controls** | | **OR (95% CI)** | **Adjusted OR** |
| --- | --- | --- | --- | --- | --- | --- |
|  | **n** | **%** | **n** | **%** |  |  |
| **All** |  |  |  |  |  |  |
| Frequent | 115 | 53.0 | 559 | 85.7 | 0.16 (0.11 to 0.24) | 0.18 (0.11 to 0.28)*** |
| No or Infrequent | 102 | 47.0 | 93 | 14.3 | Reference | Reference |
| **T category** | | | | | | |
| **T1** |  |  |  |  |  |  |
| Frequent | 87 | 64.0 | 348 | 85.5 | 0.27 (0.16 to 0.44) | 0.24 (0.13 to 0.43)**** |
| No or Infrequent | 49 | 36.0 | 59 | 14.5 | Reference | Reference |
| **T2+** |  |  |  |  |  |  |
| Frequent | 12 | 26.7 | 113 | 84.3 | 0.08 (0.03 to 0.20) | 0.09 (0.03 to 0.32)**** |
| No or Infrequent | 33 | 73.3 | 21 | 15.7 | Reference | Reference |
| **Grade** | | | | | | |
| **Well and moderate** |  |  |  |  |  |  |
| Frequent | 56 | 53.3 | 270 | 85.7 | 0.16 (0.09 to 0.30) | 0.13 (0.06 to 0.28)**** |
| No or Infrequent | 49 | 46.7 | 45 | 14.3 | Reference | Reference |
| **Poor** |  |  |  |  |  |  |
| Frequent | 40 | 50.6 | 200 | 85.1 | 0.17 (0.09 to 0.32) | 0.25 (0.11 to 0.57)**** |
| No or Infrequent | 39 | 49.4 | 35 | 14.9 | Reference | Reference |

* Frequent: at least every three years in the last ten years; infrequent: less frequently than every three years to once in the last ten years; no: no lifetime participation or no participation in the past ten years

** T category and grade apply only to cases; the controls presented are those matched to cases within these categories

*** Adjusted for all variables

**** Adjusted for education, income, number of sexual partners, body mass index and age
