# Supplementary material for: Impact of opportunistic screening on squamous cell and adenocarcinoma of the cervix in Germany: A population-based case-control study
Source: PLoS One. 2021 Jul 14;16(7):e0253801. doi: 10.1371/journal.pone.0253801 (PMC8279357; doi:10.1371/journal.pone.0253801)
Supplement: S5 Table — (DOCX) [file pone.0253801.s007.docx]

**S5 Table. Impact of cervical cancer screening on cervical cancer, according to T category, squamous cell carcinoma (172 cases and 512 controls)**

| **Participation in cervical cancer screening* by T category**** | **Cases** | | **Controls** | | **OR (95% CI)** | **Adjusted OR (95% CI)***** |
| --- | --- | --- | --- | --- | --- | --- |
|  | **n** | **%** | **n** | **%** |  |  |
| **All** |  |  |  |  |  |  |
| Frequent | 89 | 51.7 | 444 | 86.7 | 0.14 (0.09 to 0.22) | 0.14 (0.08 to 0.25) |
| No or infrequent | 83 | 48.3 | 68 | 13.3 | Reference | Reference |
| **T 1** |  |  |  |  |  |  |
| Frequent | 67 | 61.5 | 279 | 85.6 | 0.23 (0.13 to 0.40) | 0.19 (0.09 to 0.37) |
| No or infrequent | 42 | 38.5 | 47 | 14.4 | Reference | Reference |
| **T 2+** |  |  |  |  |  |  |
| Frequent | 10 | 28.6 | 90 | 86.5 | 0.07 (0.02 to 0.21) | 0.03 (0.01 to 0.25) |
| No or infrequent | 25 | 71.4 | 14 | 13.5 | Reference | Reference |

* Frequent: at least every three years in the last ten years; infrequent: less frequently than every three years to once in the last ten years; no: no lifetime participation or no participation in the past ten years

** T category applies only to cases; the controls presented are those matched to cases within these categories

*** Adjusted for education, income, number of sexual partners, body mass index and age
