# Supplementary material for: Impact of opportunistic screening on squamous cell and adenocarcinoma of the cervix in Germany: A population-based case-control study
Source: PLoS One. 2021 Jul 14;16(7):e0253801. doi: 10.1371/journal.pone.0253801 (PMC8279357; doi:10.1371/journal.pone.0253801)
Supplement: S6 Table — (DOCX) [file pone.0253801.s008.docx]

**S6 Table. Impact of cervical cancer screening on cervical cancer, according to T category, adenocarcinoma/adenosquamous cell carcinoma (40 cases and 120 controls)**

| **Participation in cervical cancer screening* by T category**** | **Cases** | | **Controls** | | **OR (95% CI)** | **Adjusted OR (95% CI)***** |
| --- | --- | --- | --- | --- | --- | --- |
|  | **n** | **%** | **n** | **%** |  |  |
| **All** |  |  |  |  |  |  |
| Frequent | 24 | 60.0 | 100 | 83.3 | 0.29 (0.12 to 0.67) | 0.24 (0.06 to 0.95) |
| No or infrequent | 16 | 40.0 | 20 | 16.7 | Reference | Reference |
| **T 1** |  |  |  |  |  |  |
| Frequent | 19 | 76.0 | 63 | 84.0 | 0.60 (0.19 to 1.88) | 0.40 (0.10 to 1.54) |
| No or infrequent | 6 | 24.0 | 9 | 12.0 | Reference | Reference |
| **T 2+** |  |  |  |  |  |  |
| Frequent | 2 | 20.0 | 23 | 76.7 | 0.12 (0.02 to 0.66) | **** |
| No or infrequent | 8 | 80.0 | 7 | 23.3 | Reference |  |

* Frequent: at least every three years in the last ten years; infrequent: less frequently than every three years to once in the last ten years; no: no lifetime participation or no participation in the past ten years

** T category applies only to cases; the controls presented are those matched to cases within these categories

*** Adjusted for education, income, number of sexual partners, body mass index and age

**** Too few cases/controls
