# Supplementary material for: Impact of opportunistic screening on squamous cell and adenocarcinoma of the cervix in Germany: A population-based case-control study
Source: PLoS One. 2021 Jul 14;16(7):e0253801. doi: 10.1371/journal.pone.0253801 (PMC8279357; doi:10.1371/journal.pone.0253801)
Supplement: S7 Table — (DOCX) [file pone.0253801.s009.docx]

**S7 Table: Factors associated with cervical cancer: univariable and multivariable conditional logistic regression, excluding women younger than 30 years (211 cases and 637 controls)**

| **Socio-demographic and risk factors** | **Univariable** | | **Multivariable*** | |
| --- | --- | --- | --- | --- |
|  | **OR** | **95% CI** | **OR** | **95% CI** |
| No or infrequent participation vs. frequent participation in cervical cancer screening [Ref.] ** | 6.57 | 4.35 to 9.91 | 5.66 | 3.50 to 9.16 |
| School education: ≥ 12 years vs < 12 years [Ref.] | 0.36 | 0.25 to 0.54 | 0.40 | 0.25 to 0.66 |
| Net monthly household income: ≥ €3000 vs.  < €3000 [Ref.] | 0.30 | 0.2 to 0.45 | 0.47 | 0.28 to 0.78 |
| Currently living with a partner: no vs yes [Ref.] | 1.89 | 1.27 to 2.82 | 0.99 | 0.59 to 1.65 |
| Parity: ≥ 4 children vs. < 4 children [Ref.] | 2.75 | 1.39 to 5.41 | 1.92 | 0.74 to 4.95 |
| Ever use of oral contraceptives: ever vs never [Ref.] | 0.56 | 0.35 to 0.91 | 0.67 | 0.35 to 1.29 |
| Number of sexual partners: ≥ 1 partner vs 1 partner [Ref.] | 3.08 | 1.77 to 5.36 | 3.01 | 1.55 to 5.82 |
| Genital herpes: ever vs never [Ref.] | 1.42 | 0.61 to 3.32 | 2.37 | 0.81 to 6.95 |
| Chlamydia: ever vs never [Ref.] | 0.81 | 0.45 to 1.44 | 0.85 | 0.40 to 1.79 |
| Condyloma: ever vs never [Ref.] | 1.14 | 0.55 to 2.34 | 1.38 | 0.56 to 3.42 |
| Smoking: ever vs never [Ref.] | 2.03 | 1.46 to 2.83 | 1.22 | 0.81 to 1.84 |
| Body Mass Index: ≥ 30 vs. < 30 kg/m^2^ [Ref.] | 1.88 | 1.25 to 2.83 | 1.69 | 1.00 to 2.86 |
| Physical activity: ≥ 30 minutes/day vs. < 30 minutes/day [Ref.] | 0.45 | 0.33 to 0.62 | 0.81 | 0.55 to 1.20 |
| Sporting activity: ≥ once a week vs < once a week [Ref.] | 1.51 | 0.77 to 2.95 | 1.64 | 0.72 to 3.76 |
| ≥ 3 portions of fruit and vegetables a day vs < 3 portions/day [Ref.] | 0.74 | 0.53 to 1.03 | 1.14 | 0.75 to 1.73 |

OR: odds ratio, CI: confidence interval, Ref: reference

* Adjusted for all variables and age

** Frequent: at least every three years in the last ten years; no or infrequent: less frequently than every three years including women who had reported no screening participation in the last ten years and women who reported no screening in their lives
